# Postabsorptive and postprandial myofibrillar protein synthesis rates at rest and after resistance exercise in women with postmenopause

**DOI:** 10.1152/japplphysiol.00886.2023

**Published:** 2024-02-22

**Authors:** Colleen F. McKenna, Andrew T. Askow, Kevin J. M. Paulussen, Amadeo F. Salvador, Hsin-Yu Fang, Alexander V. Ulanov, Zhong Li, Scott A. Paluska, Joseph W. Beals, Ralf Jäger, Martin Purpura, Nicholas A. Burd

**Affiliations:** ^1^Division of Nutritional Sciences, University of Illinois Urbana-Champaign, Urbana, Illinois, United States; ^2^Department of Kinesiology and Community Health, University of Illinois Urbana-Champaign, Urbana, Illinois, United States; ^3^Roy J. Carver Biotechnology Center, University of Illinois Urbana-Champaign, Urbana, Illinois, United States; ^4^Increnovo LLC, Milwaukee, Wisconsin, United States

**Keywords:** aging, females, leucine, protein nutrition, skeletal muscle mass

## Abstract

Feeding and resistance exercise stimulate myofibrillar protein synthesis (MPS) rates in healthy adults. This anabolic characterization of “healthy adults” has been namely focused on males. Therefore, the purpose of this study was to examine the temporal responses of MPS and anabolic signaling to resistance exercise alone or combined with the ingestion of protein in postmenopausal females and compare postabsorptive rates with young females. Sixteen females [60 ± 7 yr; body mass index (BMI) = 26 ± 12 kg·m^−2^] completed an acute bout of unilateral resistance exercise before consuming either: a fortified whey protein supplement (WHEY) or water. Participants received primed continuous infusions of L-[*ring*-^13^C_6_]phenylalanine with bilateral muscle biopsies before and after treatment ingestion at 2 h and 4 h in nonexercised and exercised legs. Resistance exercise transiently increased MPS above baseline at 0–2 h in the water condition (*P* = 0.007). Feeding after resistance exercise resulted in a late phase (2–4 h) increase in MPS in the WHEY condition (*P* = 0.005). In both conditions, resistance exercise did not enhance the cumulative (0–4 h) MPS response. In the nonexercised leg, MPS did not differ at 0–2 h, 2–4 h, or 0–4 h of the measurement periods (all, *P* > 0.05). Likewise, there were no changes in the phosphorylation of p70S6K, AMPKα, or total and phosphorylated yes-associated protein on Ser127. Finally, postabsorptive MPS was lower in premenopausal versus postmenopausal females (*P* = 0.023). Our results demonstrate that resistance exercise-induced changes in MPS are temporally regulated, but do not result in greater cumulative (0–4 h) MPS in postmenopausal women.

**NEW & NOTEWORTHY** An adequate quality and quantity of skeletal muscle is relevant to support physical performance and metabolic health. Muscle protein synthesis (MPS) is an established remodeling marker, which can be hypertrophic or nonhypertrophic. Importantly, protein ingestion and resistance exercise are two strategies that support healthy muscle by stimulating MPS. Our study shows postmenopause modulates baseline MPS that may diminish the MPS response to the fundamental anabolic stimuli of protein ingestion and resistance exercise in older females.

## Graphical abstract

**Figure:**
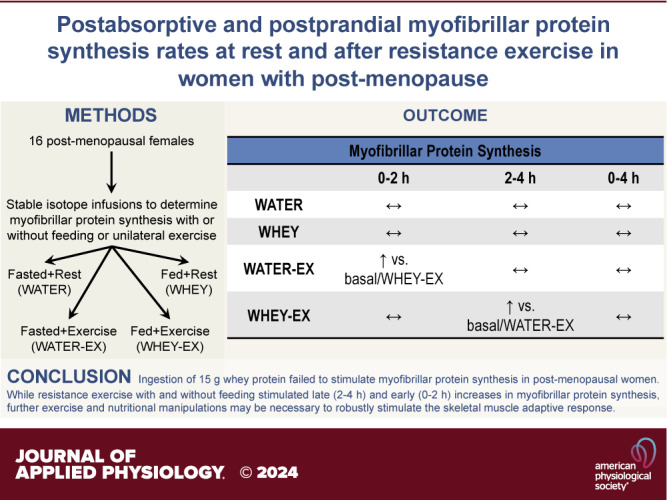

## INTRODUCTION

The aging-associated decrement in skeletal muscle mass is well characterized (1, 2). This insufficient quality and quantity of skeletal muscle mass, which results from an imbalance between muscle protein synthesis (MPS) and breakdown rates, reduces physical independence and quality of life (3, 4), and increases the risk of multimorbidities and all-cause mortality (5, 6). Acute resistance exercise and protein ingestion are two main stimuli for increasing muscle protein synthesis rates, which are generally assumed to drive subsequent changes in hypertrophic or nonhypertrophic protein remodeling in healthy adults (7). Hence, understanding the regulation of postprandial/postexercise MPS not only provides insight into the etiology of muscle protein loss but also potential anabolic strategies to enhance the quality of skeletal muscle protein in the longer term.

The majority of muscle protein turnover investigations with age have been performed in aging male participants. These male-specific observations may not translate well to aging females, partially due to divergent body composition and hormone concentrations between the sexes that may impact postabsorptive or postprandial MPS (8–13). Fortunately, however, there have been various efforts in recent years to define aging-related sex differences in postabsorptive and postprandial mixed (14–18) and fraction-specific (i.e., myofibrillar) (19) muscle protein turnover. The evidence, however, remains controversial with contrasting observations regarding sex differences in MPS with advancing age (14–17). For example, it has been shown that females, regardless of age, have higher basal mixed MPS rates than males (16), whereas other findings suggest that sex differences are only apparent with age and postmenopause (18, 20). Nevertheless, postmenopausal females typically have less muscle mass, are more susceptible to disuse-induced muscle wasting (21), and suffer from sarcopenia and musculoskeletal injuries at a greater rate in comparison to aging males (22, 23). Therefore, there is still a need to characterize the responsiveness of myofibrillar protein synthesis rates to resistance exercise, with or without protein intake, in females at a more advanced age. In fact, it has been shown that higher-intensity resistance exercise (6 sets × 8 repetitions at 75% of one-repetition maximum) does not elevate the fed-state muscle protein synthetic response in older females (24). This contradicts the hallmark characteristic of resistance exercise potentiating the fed-state response generally observed in younger (7) and older males (25). It is also important to note that information related to the anabolic action of resistance exercise alone (i.e., resistance exercise in the postabsorptive state), regardless of sex, is limited in the aging population in general (26, 27).

Therefore, we aimed to determine the early time-dependent anabolic action of resistance exercise alone (WATER) or combined with whey protein nutritional supplement (WHEY) on the stimulation of myofibrillar protein synthesis rates in postmenopausal women. In addition, as secondary outcomes, we assessed mTORC1-dependent p70 ribosomal S6 kinase (p70S6K) and mTORC1-independent yes-associated protein 1 (YAP1) anabolic signaling events (28). We hypothesized that resistance exercise would elevate rates of myofibrillar protein synthesis above the postabsorptive state regardless of nutritional status. Moreover, we hypothesized that the ingestion of WHEY would stimulate an increase in myofibrillar protein synthesis rates above postabsorptive states and resistance exercise would potentiate this response. Finally, as an exploratory outcome, we compared postabsorptive myofibrillar protein synthesis rates between young (premenopausal) (29, 30) versus postmenopausal females given the aging-associated sex-based differences in the mixed muscle protein synthetic response previously observed.

## METHODS

### Participants and Ethical Approval

Sixteen healthy postmenopausal females volunteered to participate in this study. Self-reported health questionnaires were evaluated for study inclusion. Only those 50–79 yr with a body mass index (BMI) between 18.5 and 30 kg·m^−2^, who report no chronic cardiometabolic diseases, dietary restrictions, or use of any nutritional supplements known to affect protein metabolism were considered for participation. Individuals with uncontrolled hypertension, exercise restrictions, musculoskeletal conditions or injuries sustained ≤ 1 year, or history of tobacco or marijuana use were excluded from participating in this study. The participants are part of a larger investigation defining exercise and nutritional strategies to augment the regulation of muscle mass in females.

All participants were informed of study procedures and risks associated with participation before signing an informed consent document. The protocol was approved by the Institutional Review Board at the University of Illinois Urbana-Champaign. All procedures involving human subjects were conducted in accordance with the standards for the use of human participants in research as outlined in the Declaration of Helsinki. This trial was registered at ClinicalTrials.gov as NCT02918981.

### Premenopausal Cohort

Participant characteristics of our premenopausal women cohort for the exploratory aim are shown in Table 1. Postabsorptive rates of muscle protein synthesis of young female participants (i.e., premenopausal) were taken from previous studies within our laboratory (NCT02613767 and NCT03870165). Women between the ages of 20 and 45 yr were eligible for these studies, with the same exclusion criteria as aforementioned. These participants received similar methods of determination of postabsorptive rates (primed continuous infusions of L-[*ring*-^13^C_6_]phenylalanine [*n* = 9] or L-[*ring*-^2^H_5_]phenylalanine [*n* = 5]). The young female participants performed the trials during the follicular phase of the menstrual cycle.

**Table 1.** Participant characteristics for premenopausal females

Participant Characteristics	Means ± SD
Age, yr	24 ± 3
Body mass, kg	72.0 ± 12.2
BMI, kg·m^−2^	24.9 ± 3.1
LBM, kg	47.3 ± 5.5
Fat mass, kg	22.0 ± 7.2
Body fat, %	30.0 ± 5.4
Waist:hip ratio	0.78 ± 0.07
Systolic blood pressure, mmHg	120 ± 9
Diastolic blood pressure, mmHg	75 ± 6
Fasting plasma glucose, mmol·L^−1^	4.3 ± 0.2
Fasting plasma insulin, µIU·mL^−1^	5.9 ± 2.3

Data are means ± SD. BMI, body mass index; LBM, lean body mass.

### Study Overview and Experimental Design

The postmenopausal women study utilized a randomized controlled, parallel-group design. We used a unilateral study approach whereby participants completed single-leg exercise, while their rested leg serves as a nonexercise control to allow for rest and exercise responses to be compared within the same individual and, as such, increased statistical power (29). Moreover, we sought to assess the effects of a whey protein nutritional supplement (WHEY) compared with a water control (WATER) on the postprandial regulation of myofibrillar protein synthesis rates in a time-dependent manner at early (0–2 h), later (2–4 h), or cumulative (0–4) postprandial/postexercise periods.

### Screening

Participants arrived to the laboratory in the morning after an overnight (≥12 h) fast having refrained from strenuous exercise, alcohol consumption, and caffeine intake for 72 h, 48 h, and 24 h, respectively, to complete screening procedures. Upon arrival, participants were seated upright with their legs uncrossed for at least 5 min before measurement of resting blood pressure. Subsequently, body mass, height, and waist-to-hip ratio were measured before determination of body composition via dual-energy X-ray absorptiometry (Hologic QDR 4500 A, Bedford, MA). Following the determination of body composition, participants underwent 12-repetition maximum (12RM) testing for unilateral leg extension to estimate 1RM as previously described (31). In brief, unilateral leg extension was assessed in the participant’s dominant leg using a guided-motion unilateral leg extension machine. A repetition was deemed successful when the participant was able to move the weight through the full range of motion as judged by a trained member of the research team. The 12RM was determined within three attempts with 3 min rest allowed between attempts. The machine settings were recorded for each participant to ensure proper placement for the experimental infusion. The estimated 1RM was used to determine working load for the experimental exercise bout.

### Dietary and Exercise Control

For all participants (both post- and premenopausal studies), experimental infusions were performed at least 72 h, but no more than 1 wk, following completion of the screening trial. Before the experimental infusion, participants were instructed to refrain from strenuous exercise for 72 h, alcohol consumption for 48 h, and food or beverages (with the exception of water) for 12 h. For the 48 h prior, all participants were instructed to consume and record their dietary intakes using the Automated Self-Administered 24-H (ASA24) Dietary Assessment Tool (v. 2016; National Cancer Institute, Rockville, MD). Participants were also provided with a standardized meal to be ingested the night before the infusion.

### Treatment Beverage

The nutritional supplement (WHEY) was provided in powdered form (Curves Japan Co., Ltd., Tokyo, Japan) and contained whey protein isolate that was fortified with vitamins (C, B1, B2, B3, B5, B6, and B12) and minerals (calcium, magnesium, iron, zinc, and potassium). The whey protein was dosed relative to lean body mass (LBM) at 0.29 g·kg·LBM^−1^. The average protein bolus for the WHEY group was 12.8 ± 1.7 g. The nutritional supplement was dissolved in 250 mL of water. To minimize disturbances in precursor pool isotopic equilibrium, the nutritional supplement was enriched to approximately 6% with tracer according to the phenylalanine content of approximately 3.5% in the whey protein, which equals to 1.568 mg L-[*ring*-^13^C_6_]phenylalanine·g·protein^−1^ (32). Those randomized to the WATER condition ingested 250 mL of water. The protein dosing is consistent with amounts recommended within the 2020–2025 Dietary Guidelines for Americans (DGA). Specifically, this protein dosing amount is comparable to approximately 2 ounce-equivalents (oz-equiv) of protein foods within the DGA MyPlate framework, which recommends 5–6 oz-equiv daily for middle-aged (31–59 yr) and older-aged women (>60 yr) (33). As such, this approach allows us to comment on the effectiveness of DGAs for the regulation of muscle mass and improved translation for nutrition and public health messaging.

### Infusion Protocol

**Figure 1.**
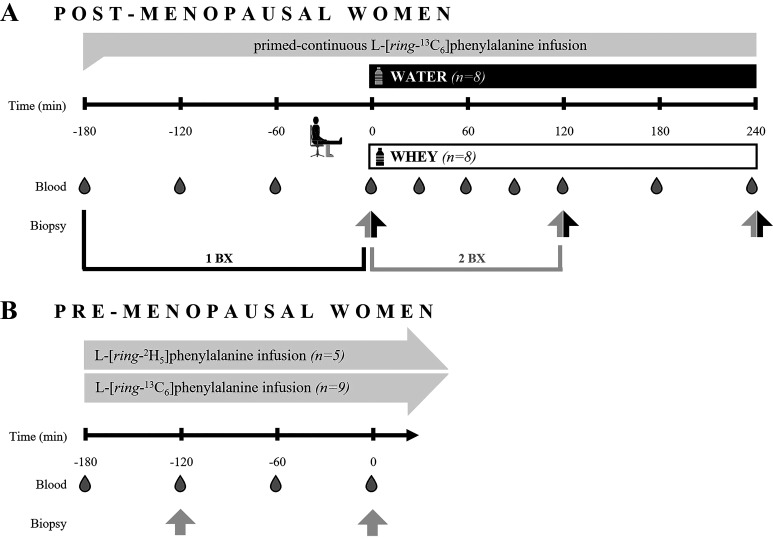
Schematic overview of the infusion protocols. *A*: the study in postmenopausal women included ingestion of the treatment beverage (parallel groups: WHEY or WATER) immediately after unilateral leg extension exercise. Bilateral muscle biopsies were collected from the exercised (black arrow) and rest (gray arrow) legs. The single biopsy (1BX) and sequential biopsy (2BX) used ^13^C enrichments from the baseline (−180 min) blood sample to rest leg biopsy (0 min), and 0–120 min rest leg biopsies, respectively. *B*: postabsorptive biopsies in premenopausal women were over a 2-h rested/fasted period after 1 h incorporation of respective stable isotope. Blood samples were collected throughout the experiment. WATER, water control; WHEY, whey protein supplement.

An overview of experimental infusions is shown in Fig. 1. On the morning of all trials, participants arrived to the laboratory by car or public transportation following an overnight fast. Upon arrival, an intravenous catheter was inserted into a dorsal hand or antecubital vein before baseline blood collection (*t* = −180 min). For the postmenopausal study (Fig. 1*A*), a primed-continuous infusion of L-[*ring*-^13^C_6_]phenylalanine [prime: 2 µmol·kg·lean body mass (LBM)^−1^; continuous: 0.05 µmol·kg·LBM^−1^·min^−1^] was initiated and maintained until the end of the experiment (*t* = 240 min). A second intravenous catheter was placed in the contralateral heated dorsal hand or antecubital vein and kept patent by a 0.9% saline drip for repeated arterialized blood sampling. Participants performed an acute bout of unilateral resistance exercise on a guided-motion leg extension machine at *t* = −30 min of the infusion protocol. The exercise bout started with a warm-up (2 sets of 12 repetitions at 30% of 1RM) before completing 3 sets of 12 repetitions at 65% 1RM with 90 s of rest provided between sets. After the exercise bout (*t* = 0 min), bilateral muscle biopsies were collected from the middle region of the m. vastus lateralis with a Bergström needle modified for manual suction under local anesthetic (2% xylocaine). Muscle tissue was isolated from visible blood, adipose, and connective tissue, and snap frozen in liquid nitrogen before storage at −80°C until analysis. Immediately following the 0-min biopsy, participants ingested their randomized treatment beverage (WATER or WHEY). Additional bilateral biopsies were collected 120 and 240 min following consumption of WATER or WHEY. Blood samples were collected throughout the experiment according to Fig. 1 in ethylenediaminetetraacetic acid (EDTA)-coated vacutainers and centrifuged at 3,000 *g* for 10 min at 4°C. Plasma was aliquoted and stored at −80°C for future analyses.

The infusion protocols from which our data on premenopausal women is sourced are detailed elsewhere (29, 30), with an overview of the postabsorptive time course depicted in Fig. 1*B.* Two muscle biopsies were sampled as aforementioned, for the determination of postabsorptive muscle protein synthesis by the sequential biopsy approach.

### Blood Analysis

Whole blood glucose was assessed with an automated biochemical analyzer (YSI 2900, Yellow Springs, OH). Plasma insulin, progesterone, 17β-estradiol, free testosterone, and sex hormone-binding globulin (SHBG) concentrations were measured with commercially available enzyme-linked immunosorbent assays (insulin: 80-INSHU-E01.1 ALPCO Diagnostics, Salem, NH; progesterone: IB79105 Immuno-Biological Laboratories, Inc., Minneapolis, MN; estradiol: ab108667, free testosterone: ab178663 SHBG: ab213824 Abcam, Cambridge, UK). Plasma amino acid concentrations were measured as previously described (30). Briefly, plasma samples (50 µL) were spiked with 10 µL of internal standard (DL-p-chlorophenylalanine; 1 mg·mL^−1^ in 0.1 M HCl) and deproteinized with 940 µL methanol. Subsequently, samples were spun at 20,817 *g* for 10 min at 4°C, and 700 µL of supernatant was transferred to a clean, dry tube for evaporation under vacuum. Dehydrated samples were resuspended in 0.1% formic acid for the determination of amino acid concentrations via liquid chromatography tandem mass spectrometry (LC/MS/MS). The calibration curve was created with an amino acid standard solution (AAS18, Sigma-Aldrich, St. Louis, MO) and a custom mixture for l-tryptophan, L-glutamine, l-asparagine, L-citrulline, and l-cysteine. Internal standard (ε-aminocaproic acid mg·mL^−1^) was added to each sample and standard solution. Selected reaction monitoring was used for the amino acid quantitation.

### Myofibrillar Protein Synthesis Analysis

All muscle biopsies from the postmenopausal study and postabsorptive muscle biopsies from the premenopausal cohort were analyzed for myofibrillar protein synthesis. Myofibrillar protein‐enriched fraction was extracted as previously described (28). Wet muscle (∼50 mg) was homogenized with ice-cold radioimmunoprecipitation buffer (10 μL·mg·tissue^−1^) supplemented with protease and phosphatase inhibitor tablets (cOmplete and PhosSTOP, Roche Applied Science, Mannheim, DEU). Following homogenization, samples were spun at 700 *g* for 15 min at 4°C, and the supernatant was removed and stored at −80°C for subsequent Western blotting (described in *Immunoblotting*). The myofibrillar-enriched pellet was washed in *dd*H_2_O and solubilized in 1 mL of 0.3 M NaOH at 37°C for 30 min with vortexing every 10 min. After solubilizing, samples were spun at 10,000 *g* for 5 min at 4°C, and the supernatant was transferred to glass vials. Another 1 mL of 0.3 M NaOH was added to the remaining pellet, vortexed for 10–15 s, and centrifuged.

Following transfer of the supernatant to the same glass vial, solubilized myofibrillar proteins were precipitated using 1 mL of 1 M perchloric acid and centrifuged at 3,000 *g* for 10 min at 4°C. After discarding the supernatant and washing proteins in 500 µL of 70% ethanol, samples were hydrolyzed overnight in 1.5 mL 6 M HCl at 110°C. The resultant free amino acids were purified using cation exchange chromatography (Dowex 50 W‐X8‐200 resin; Acros Organics, Geel, BEL), dried under vacuum, resuspended in 0.1 M HCl, then analyzed by 5500 QTRAP (Sciex, Redwood City, CA) LC/MS/MS as previously described (28). L‐[*ring*‐^13^C_6_]phenylalanine enrichments (tracer-tracee ratio, TTR) were determined by multiple reaction monitoring at mass-to-charge ratio (*m/z*) 166.0 → 103.0 and 172.0 → 109.0 for unlabeled and labeled L‐[*ring*‐^13^C_6_]phenylalanine, respectively. Analyst V1.6.2 (Sciex) was used for data acquisition and analysis.

### Calculations

The fractional synthetic rate (FSR) of myofibrillar proteins was calculated using the standard precursor product equation:

$$\text{FSR }\left(\% \times {\text{h}}^{-1}\right)=\left(\frac{\Delta E_{\text{protein bound}}}{E_{\text{precursor }}\times \Delta t}\right)\times 100\%$$

Although △*E*_protein bound_ (Ep2 − Ep1) is the change in myofibrillar protein enrichment over time (t), *E*_precursor_ refers to the weighted mean of plasma enrichment over time. The recruitment of tracer-naive participants allowed us to use the preinfusion blood sample (i.e., mixed plasma protein) as the baseline enrichment, commonly referred to as the single biopsy approach (32, 34), for the calculation of postabsorptive FSR from rest leg (0 min) as previously described (32).

For the premenopausal versus postmenopausal basal FSR comparison, we made comparisons based on both the single biopsy (1BX) and sequential biopsy approach (2BX) (Fig. 1*A*). We did this because the single-biopsy approach is considered to provide a reference FSR value as opposed to a “true” estimate of the baseline muscle protein synthetic value (35). Briefly, are the myofibrillar protein-bound enrichments measured directly at 0 (Ep1) and 2 h (EP2) of the infusion for 2BX postmenopausal and premenopausal with incorporation times of 2.23 ± 0.09 h and 2.02 ± 0.09 h, respectively. For the 1BX postmenopausal condition, Ep1 is the myofibrillar protein-bound enrichments measured in the biopsy collected with an incorporation time of 3.45 ± 0.22 h (*t* = 0 min), and Ep2 is the mixed plasma protein enrichment at baseline (*t =* −180 min).

### Immunoblotting

Western blots were performed as previously described (28). Total protein concentration of muscle homogenate was determined by bicinchoninic acid (BCA) assay (Pierce, Rockford, IL). Equal amounts of protein from each sample were diluted 1:1 with 2× Laemmli (MilliporeSigma, St. Louis, MO), denatured at 95°C for 5 min, then separated on 7.5% or 10% (wt/vol) polyacrylamide gels by electrophoresis. Separated proteins were transferred to polyvinylidene fluoride membranes (PVDF, MilliporeSigma, St. Louis, MO). Membranes were blocked with nonfat milk or bovine serum albumin diluted in Tris-buffered saline with Tween solution (TBS-T) for 1 h at room temperature before overnight incubation in primary antibodies at 4°C. Proteins of interest were detected with primary antibodies as listed in Supplemental Table S1. After primary incubation, blots were exposed to secondary antibody of corresponding animal and detected by ECL Western blotting substrate (Thermo Scientific, Waltham, MA) with the ChemiDoc XRS+ Imaging System (Bio-Rad Laboratories, Hercules, CA). Bands were quantified using ImageJ (National Institute of Health, Bethesda, MD) and normalized to both α-tubulin as the internal control and a control sample included on each blot to account for interblot variability.

### Statistical Analysis

All data are presented as means ± SD and/or mean difference with 95% confidence intervals (CI) where appropriate. Participant characteristics, reported dietary intake, and exercise variables were analyzed with an independent samples *t* test for group comparisons. Differences in blood analytes were assessed using linear mixed-effects models with group and time as fixed factors. Differences in myofibrillar FSR and anabolic signaling were assessed via linear mixed-effects models with leg, time, and condition as fixed factors. Bonferroni post hoc comparisons were calculated when significant main effects or interactions were detected. All analyses were conducted using IBM Statistics for Windows (v. 28.0.1.0; IBM Corp., Armonk, NY). Differences were considered statistically significant when *P* < 0.05.

## RESULTS

### Participants

Participant characteristics for primary outcomes are displayed in Table 2. There were no differences between WHEY and WATER conditions in baseline characteristics. Moreover, reported nutrient intake during the 48 h preceding the experimental infusion was not different between WHEY and WATER conditions (Table 3). Muscular strength as measured by dominant leg extension 12RM was not different between groups (WHEY: 11.1 ± 3.1 kg, WATER: 8.9 ± 3.3 kg, *P* = 0.232). During the experimental infusion, volume load of the unilateral leg extension exercise bout was not different between groups (WHEY: 427 ± 116 kg, WATER: 377.6 ± 130 kg, *P* = 0.434).

**Table 2.** Participant characteristics of postmenopausal cohort

Participant Characteristics	WHEY (*n* = 8)	WATER (*n* = 8)	*P* Value
Age, yr	60 ± 7	60 ± 8	0.950
Body mass, kg	71.9 ± 7.8	71.3 ± 15.0	0.922
BMI, kg·m^−2^	26 ± 2	26 ± 4	0.849
LBM, kg	44.4 ± 6.1	42.3 ± 6.0	0.501
Fat mass, kg	25.5 ± 6.3	27.4 ± 10.8	0.673
Body fat, %	35.2 ± 7.3	37.3 ± 6.9	0.558
Waist:hip ratio	0.86 ± 0.10	0.81 ± 0.06	0.348
Systolic blood pressure, mmHg	118 ± 15	125 ± 8	0.373
Diastolic blood pressure, mmHg	74 ± 11	78 ± 3	0.410
Fasting plasma glucose, mmol·L^−1^	4.4 ± 0.2	4.4 ± 0.8	0.820
Fasting plasma insulin, µIU·mL^−1^	6.8 ± 4.8	9.1 ± 6.6	0.477
Progesterone, ng·mL^−1^	0.529 ± 0.162	0.391 ± 0.087	0.088
Estradiol, pg·mL^−1^	18.26 ± 18.21	30.27 ± 15.74	0.069
Free testosterone, ng·dL^−1^	0.052 ± 0.040	0.020 ± 0.017	0.194
SHBG, nmol·L^−1^	81.47 ± 41.47	55.54 ± 21.30	0.145

Data are means ± SD. BMI, body mass index; LBM, lean body mass; SHBG, sex hormone-binding globulin; WATER, water control; WHEY, whey protein supplement.

**Table 3.** Self-reported dietary intakes for the 2 days preceding infusion trials

Reported Nutrient Intake	WHEY (*n* = 8)	WATER (*n* = 8)	*P* Value
Energy, kcal·day^−1^	1,609 ± 479	1,966 ± 966	0.405
Carbohydrate, g·day^−1^	175 ± 50	245 ± 115	0.161
Fat, g·day^−1^	63 ± 30	76 ± 49	0.552
Protein, g·day^−1^	76 ± 27	80 ± 22	0.730
Relative protein, g·kg^−1^·day^−1^	0.99 ± 0.35	1.05 ± 0.29	0.730

Data are means ± SD. WATER, water control; WHEY, whey protein supplement.

### Glycemic Response

**Figure 2.**
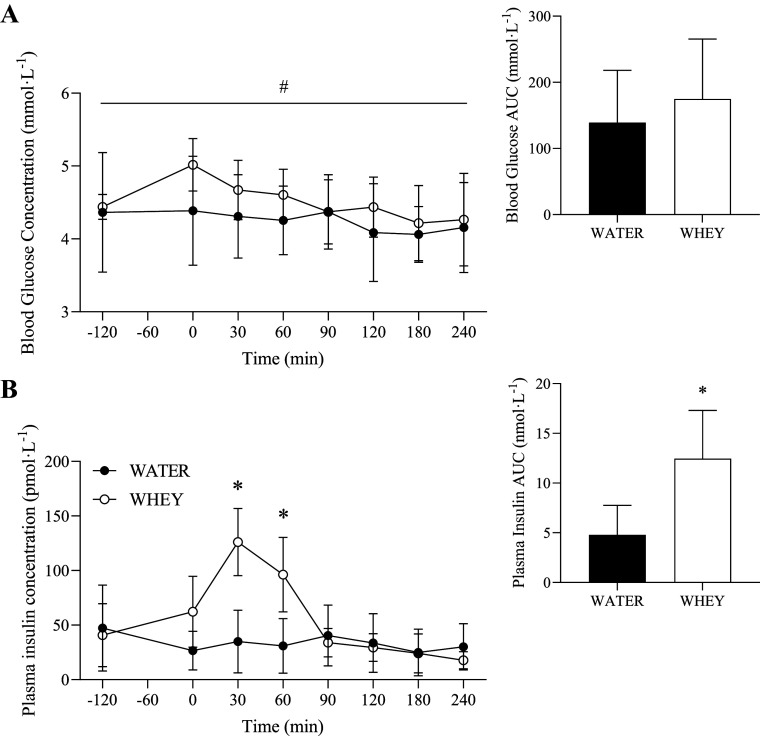
Blood glucose (*A*) and plasma insulin (*B*) concentrations before and following ingestion of WHEY (open circles) or WATER (filled circles) in postmenopausal women. Data shown as means ± SD. *Insets* show area under the curve (AUC). #Significant (*P* < 0.05) main effect of condition. *Significant (*P* < 0.05) difference between WHEY and WATER at a given time point. WATER, water control; WHEY, whey protein supplement.

Plasma glucose and insulin concentrations during the experimental infusion are shown in Fig. 2. There was a main effect of condition for plasma glucose concentration with no effect of time (*P* = 0.268) or condition × time interaction (*P* = 0.968; Fig. 2*A*). Regardless of time point, blood glucose was higher for WHEY compared with WATER (mean difference [95% CI]; 0.25 [0.07, 0.44] mmol·L^−1^, *P* = 0.007). Moreover, no differences in area under the curve (AUC) were observed for plasma glucose (*P* = 0.469).

There was a significant main effect of time (*P* = 0.002), condition (14.04 [1.82, 26.26] pmol·L^−1^, *P* = 0.025), and group × time interaction (*P* = 0.004) for plasma insulin concentrations. Plasma insulin was significantly elevated in WHEY compared with WATER condition at *t* = 30 and 60 min (Fig. 2*B*). Plasma insulin AUC was significantly higher for WHEY compared with WATER condition (*P* = 0.006).

### Plasma Amino Acids

**Figure 3.**
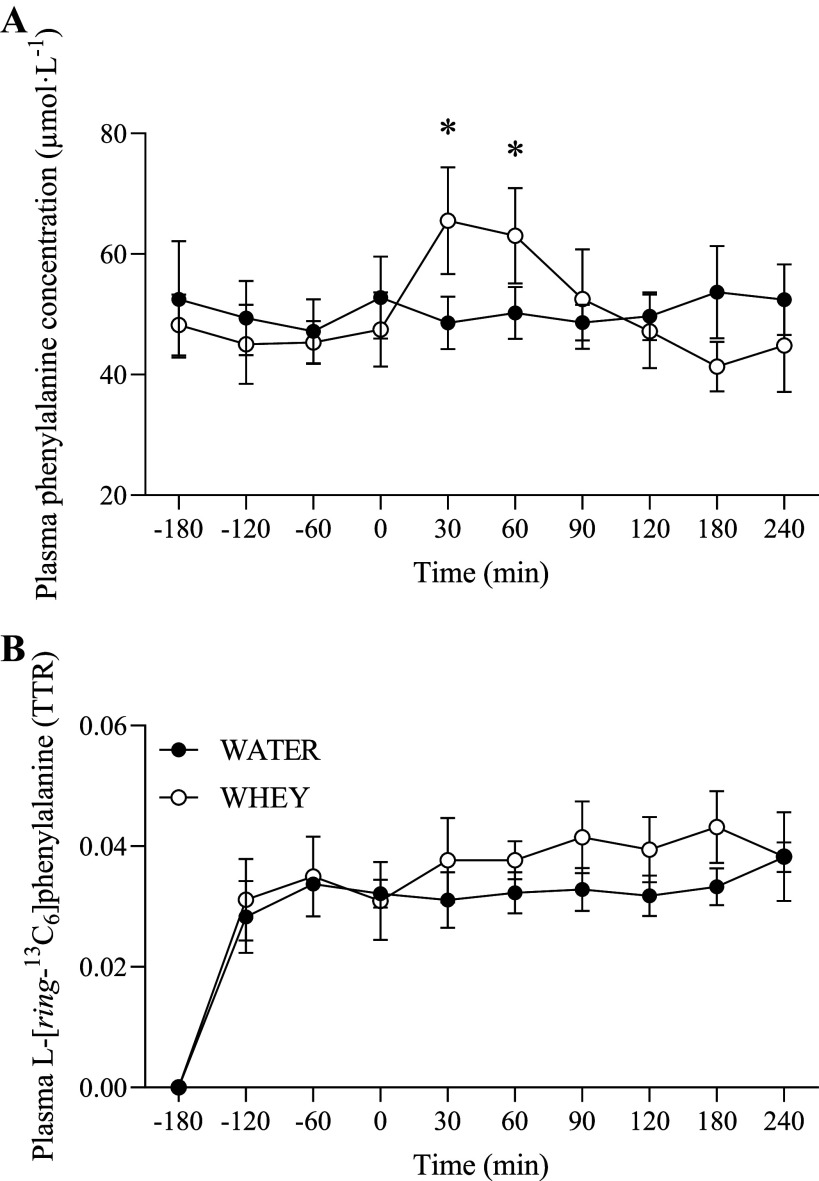
Plasma phenylalanine concentrations (*A*) and enrichments (*B*) before and following ingestion of WHEY (open circles) or WATER (filled circles) in postmenopausal women. Data shown as means ± SD. *Significant difference between WHEY and WATER at a given time point. WATER, water control; WHEY, whey protein supplement.

**Figure 4.**
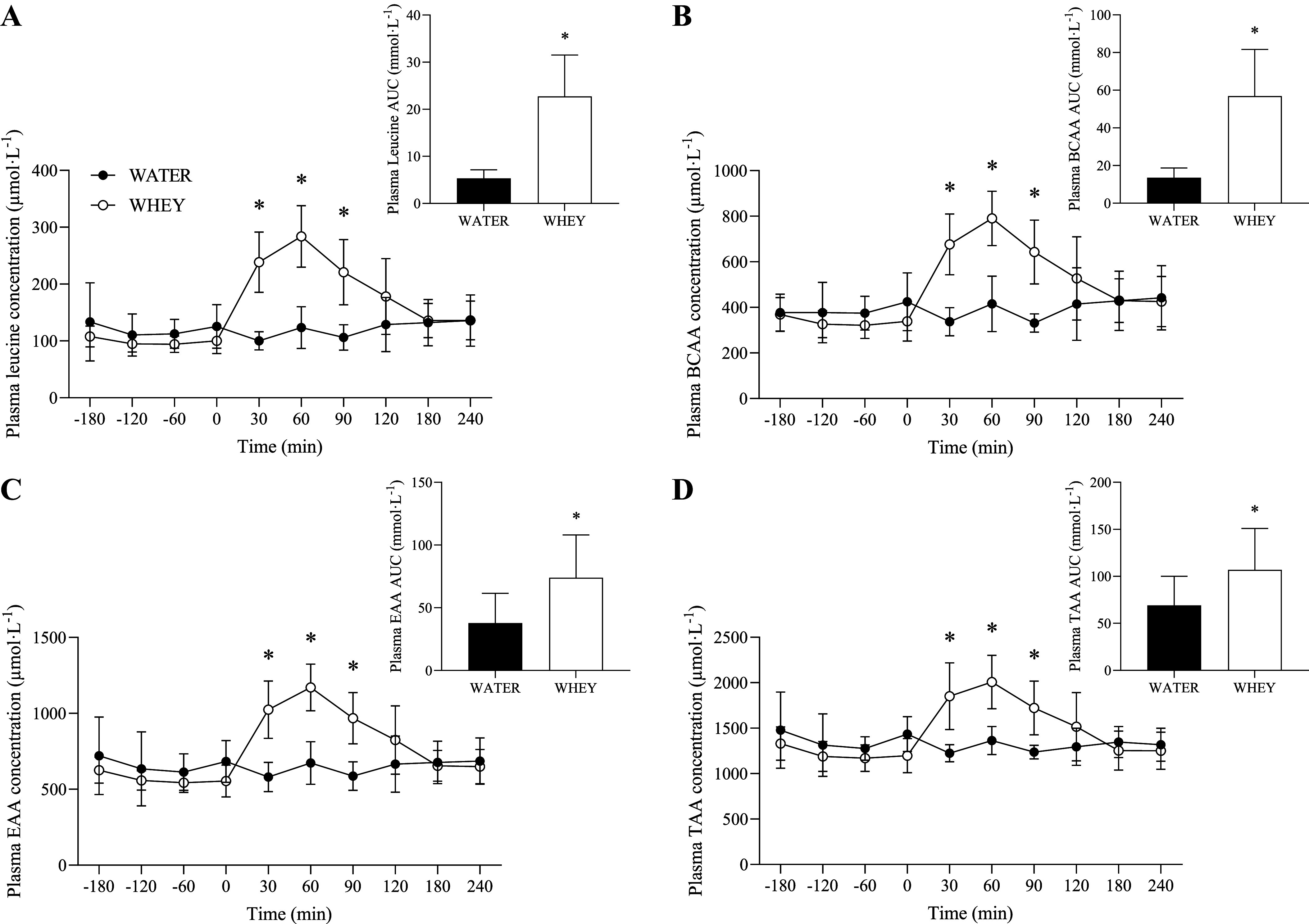
Plasma leucine (*A*), branched-chain amino acid (BCAA; *B*), essential amino acid (EAA; *C*), and total amino acid (TAA; *D*) concentration before and following ingestion of WHEY (open circles) or WATER (filled circles) in postmenopausal women. Data shown as means ± SD. *Insets* show area under the curve (AUC). *Significant difference between WHEY and WATER at a given time point. WATER, water control; WHEY, whey protein supplement.

Plasma phenylalanine concentrations and enrichments are shown in Fig. 3. A significant group × time interaction was observed for phenylalanine concentrations (*P* < 0.001). Plasma phenylalanine concentrations were significantly elevated above baseline at *t* = 30 and 60 min for WHEY but no elevations were observed for WATER. Plasma amino acids concentrations throughout the experimental infusion are depicted in Fig. 4. A significant group × time interaction was observed for plasma concentrations of leucine, branched-chain amino acids (BCAA), essential amino acids (EAA), and total amino acids (TAA; all *P* < 0.001). Plasma concentrations of leucine (Fig. 4*A*), BCAA (Fig. 4*B*), EAA (Fig. 4*C*), and TAA (Fig. 4*D*) did not change over time for WATER but were significantly elevated over baseline and WATER at *t* = 30, 60, and 90 for WHEY. Moreover, AUC was significantly greater after ingestion of WHEY compared with WATER for plasma leucine (*P* < 0.001), BCAAs (*P* = 0.001), EAAs (*P* = 0.028), and TAAs (*P* = 0.048).

### Anabolic Signaling

**Figure 5.**
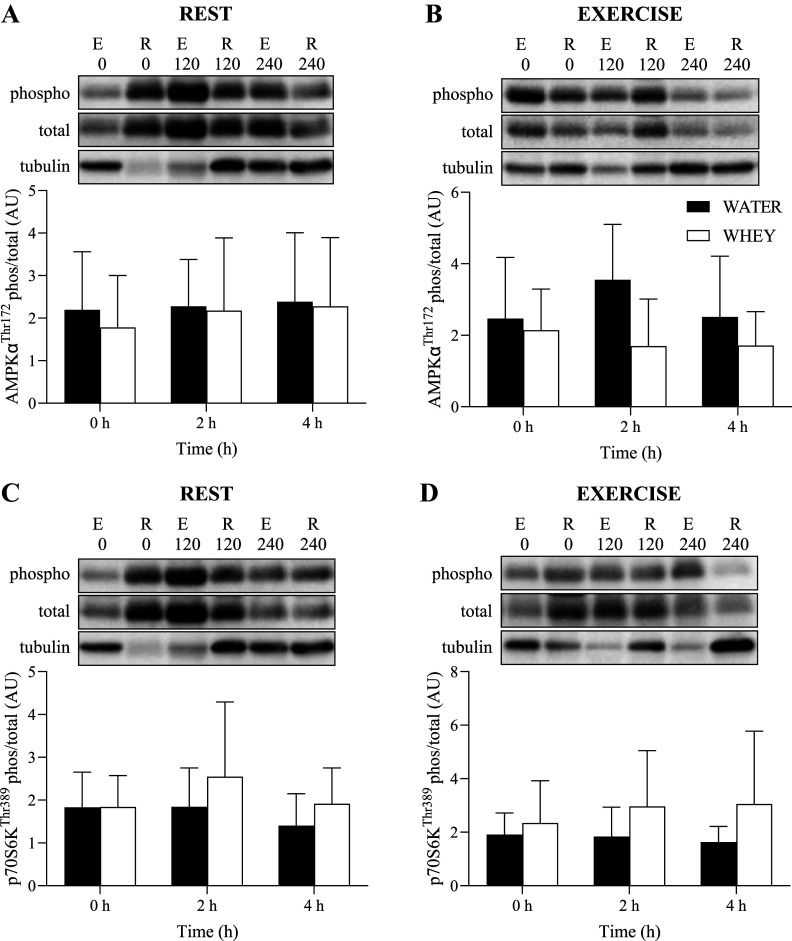
Phosphorylated-to-total ratio (normalized to α-tubulin) of AMPKα^Thr172^ for the rest (*A*) and exercised (*B*) leg and phosphorylated-to-total ratio (normalized to α-tubulin) of p70S6K^Thr389^ for the rest (*C*) and exercise (*D*) leg following ingestion of WHEY (open bars) or WATER (filled bars) in postmenopausal women. Data shown as means ± SD. WATER, water control; WHEY, whey protein supplement.

**Figure 6.**
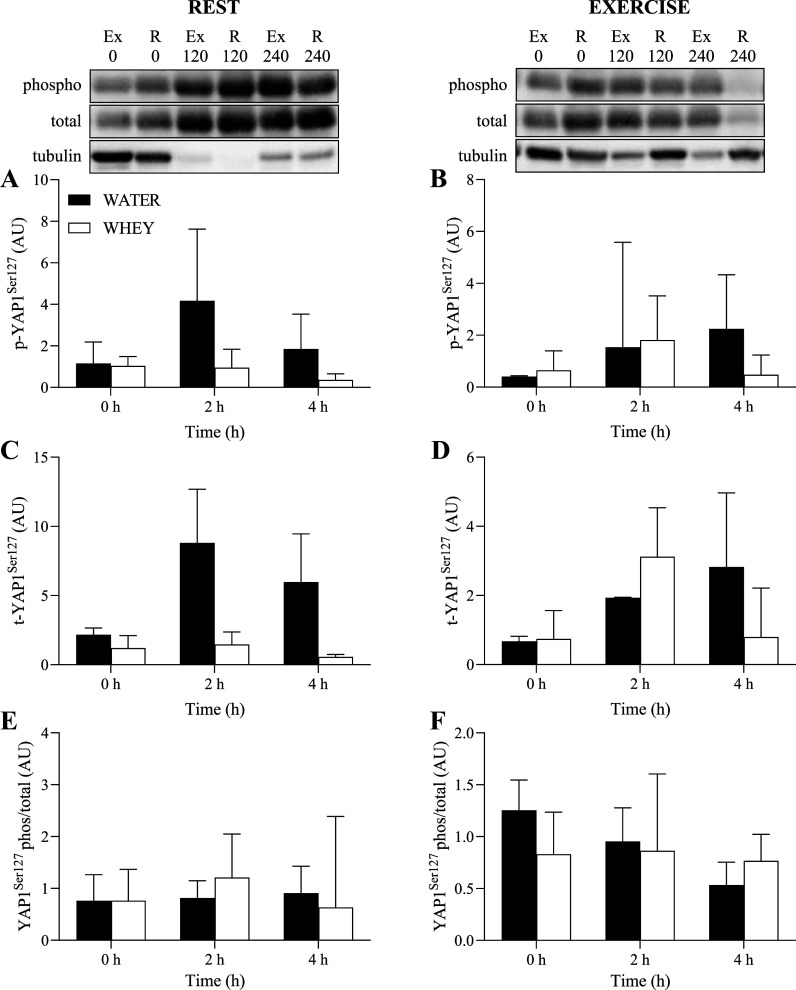
Phosphorylated (*A* and *B*), total (*C* and *D*), and phosphorylated-to-total ratio (*E* and *F*) of YAP1 for the rest (*A*, *C*, and *E*) and exercised (*B*, *D*, and *F*) leg (all normalized to α-tubulin) following ingestion of WHEY (open bars) or WATER (filled bars) in postmenopausal women. Data shown as means ± SD. WATER, water control; WHEY, whey protein supplement; YAP1, yes-associated protein 1.

Phosphorylation of anabolic signaling targets normalized to α-tubulin is presented in Figs. 5 and 6. There were no significant main effects for time, exercise, or group, nor any significant interactions, for phosphorylation of YAP1^Ser127^, p70S6K^Thr389^, or AMPKα^Thr172^.

### Myofibrillar Protein Synthesis

**Figure 7.**
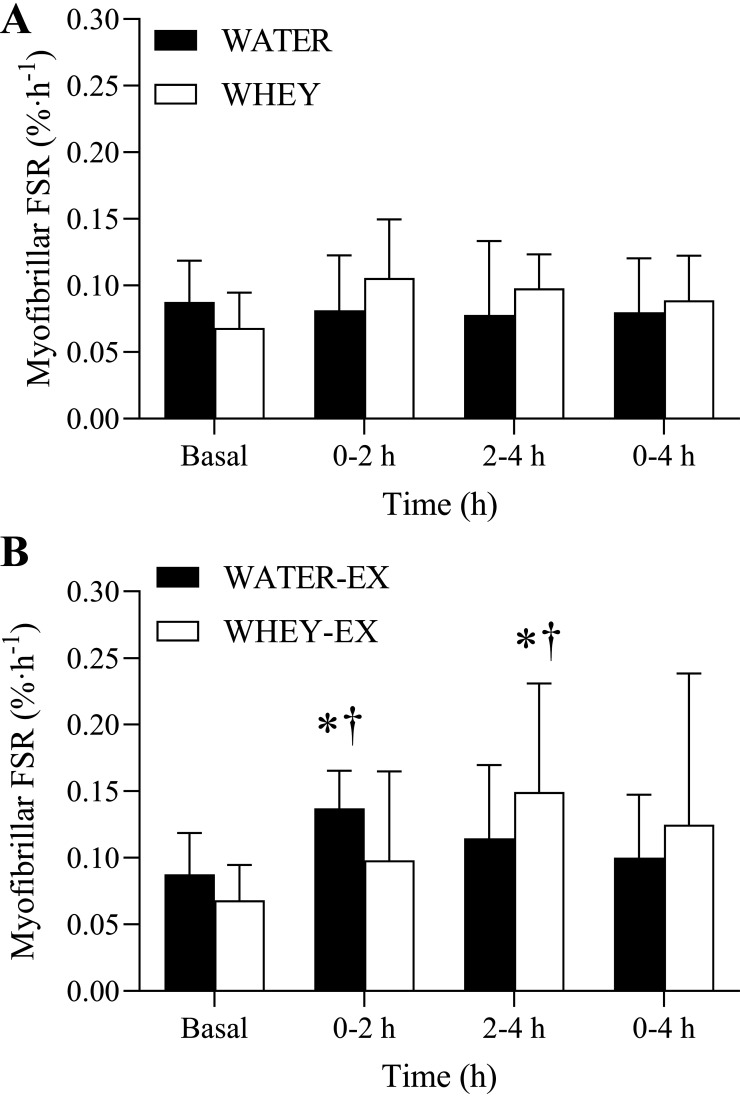
Myofibrillar fractional synthesis rates (FSRs) for the rest (*A*) and exercised (*B*) leg before and following the ingestion of WHEY (open bars) or WATER (filled bars) in postmenopausal women. Data shown as means ± SD. *Significant (*P* < 0.05) effect of leg (rest vs. exercised leg; †significant (*P* < 0.05) elevation above basal rates. WATER, water control; WHEY, whey protein supplement.

There were no differences in the myofibrillar protein synthesis rates in the resting leg of the water and WHEY conditions (Fig. 7*A*). Resistance exercise alone stimulated an increase in the myofibrillar protein synthetic response above basal at 0–2 h of the recovery period (Fig. 7*B*; *P* = 0.007), and this response was greater compared with the resting leg at this same time point (*P* = 0.008). There was a tendency for resistance exercise alone to increase the cumulative myofibrillar protein synthetic response at 0–4 h of recovery (*P* = 0.090). The ingestion of whey protein after resistance exercise stimulated a late phase increase in myofibrillar protein synthesis at 2–4 h (*P* = 0.005), and this response was greater than the resting leg at this same time point (*P* = 0.033).

### Pre- versus Postmenopausal FSR Comparison

**Figure 8.**
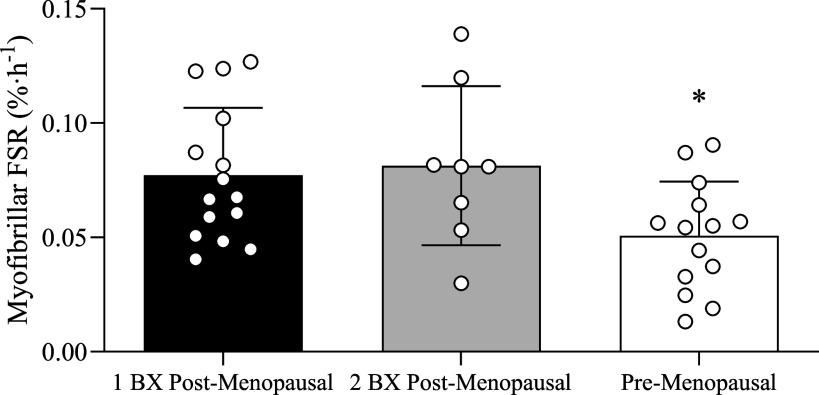
Myofibrillar fractional synthesis rates (FSRs) calculated during postabsorptive conditions by using the plasma-free tracer enrichment as the precursor pool and using mixed plasma protein enrichment and a muscle biopsy enrichment at 3.45 ± 0.22 h (1BX) or sequential muscle biopsy enrichments (2BX). Comparisons of basal myofibrillar protein synthesis rates between pre- vs. postmenopausal are also shown. Data shown as means ± SD. *Significant (*P* < 0.05) difference from 1BX and 2BX calculation of postabsorptive myofibrillar protein synthesis rates in postmenopausal females.

Participant characteristics of our premenopausal cohort for the exploratory aims are presented in Table 3. Postabsorptive myofibrillar protein synthesis rates were lower in premenopausal females compared with postmenopausal females (Fig. 8; *P* = 0.023).

### Single- versus two-Biopsy Approach

Myofibrillar protein synthesis rates were not different between 1BX and 2BX approaches (Fig. 8; *P* = 0.763).

## DISCUSSION

Resistance exercise and protein ingestion have been shown to act separately and together to increase muscle protein synthesis rates in healthy young males (7), young females (7), and older men (36); far less information exists in postmenopause. In fact, the postexercise regulation of myofibrillar protein synthesis rates in response to resistance exercise alone (in absence of nutritional intake) has not been studied in post menopause. Here, we show that resistance exercise alone stimulates a transient increase in the myofibrillar protein synthetic rates in the 0–2 h time period that returns to baseline by 2–4 h. This short-term increase did not manifest into a significant cumulative (0–4 h) myofibrillar protein synthetic response. In contrast, whey protein ingestion immediately after resistance exercise stimulated a late-phase (2–4 h) increase in the myofibrillar protein synthetic response with no impact on the cumulative myofibrillar protein synthetic response.

Past efforts have shown a blunted, yet still modifiable, anabolic response of myofibrillar protein synthesis rates to resistance exercise alone in older compared with younger males (26, 27). Specifically, older males have demonstrated a lesser myofibrillar protein synthetic response to resistance exercise (27), which is able to be restored to a more “youthful” anabolic response with increased resistance exercise volume load (6 sets > 3 sets) (27). Here, we used an exercise prescription of 3 sets of 12 repetitions at 65% 1-RM (+ warm-up sets), which is generally consistent with public health recommendations for resistance training for older adults (37, 38). Hence, it could be speculated that a more robust exercise prescription (i.e., manipulation of volume load) may be required to elicit a sustained postexercise myofibrillar protein synthetic response in postmenopausal females as previously suggested in older males (26). This is consistent with the notion that exercise volume load is an important regulatory factor for both the acute amplitude and duration of the postexercise muscle protein synthetic response (39), which may be even more true for anabolically resistant aging muscle.

It has been shown that aging muscle also demonstrates a reduced response of muscle protein synthesis rates to protein feeding (40), and this anabolic resistance may be more capparent in postmenopausal females (18). Here, we show that the ingestion of approximately 12 g of whey protein did not stimulate a rise in myofibrillar protein synthesis rates at any time point of the postprandial period (Fig. 7*A*). Interestingly, exercise before food intake was able to stimulate a late phase rise in the myofibrillar protein synthetic response at 2–4 h with no increase in the cumulative (0–4 h) myofibrillar protein synthetic response (Fig. 7*B*). Interestingly, this delayed stimulation of muscle protein synthesis rates to the anabolic action of dietary amino acids and resistance exercise has been previously observed in older males (41). The mechanistic underpinnings of this delayed feeding and resistance exercise-mediated anabolic response have yet to be clearly defined; however, in our hands, this stimulation of myofibrillar protein synthesis rates occurred after peak postprandial plasma amino acid concentrations occurred (Fig. 4). Indeed, past efforts have shown that the ingestion of 15 g of whey protein, and the subsequent postprandial aminoacidemia, was an insufficient stimulus to elicit a rise in the stimulation of postexercise myofibrillar protein synthesis rates when measured over a 0–3 h incorporation window in postmenopausal women during energy restriction (42). This suspended stimulation of the myofibrillar protein synthetic response in our study perhaps suggests that the postprandial measurement length is an important consideration in the determination of the anabolic regulation of muscle in postmenopausal females. In any case, future studies are required to better define the mechanisms that contribute to this suspended postexercise/postprandial anabolism, which may relate to elevated basal muscle protein turnover, diminished mechanosensitive pathways, and/or impaired ribosomal activity/translational efficiency and capacity (43). Importantly, our design also allows us to assess the responses to exercise alone (i.e., in absence of nutritional intake) compared with the combined effects of exercise and feeding. In contrast to younger adults (7, 44, 45) and older men (25), we did not observe the hallmark interactive effect of feeding and resistance exercise on muscle anabolic responses. Again, this may relate to inadequate exercise prescription (e.g., requirement of greater training volume for aging muscle) (27) or perhaps an intrinsic issue with the anabolic sensitivity of myofibrillar protein synthesis due to dysregulated basal muscle protein synthesis rates in postmenopausal women (46).

The anabolic sensitivity of mixed muscle protein synthesis rates to anabolic stimuli has been shown to be inversely related to basal muscle protein turnover rates (i.e., higher basal muscle protein synthesis associated with reduced responsiveness) (46). Moreover, other works have demonstrated an elevated basal mixed muscle protein synthetic response in older females compared with older males (18, 47). This sex-based difference in basal muscle protein synthesis rates is not present when comparing younger males and females (48). Here, we compared basal myofibrillar protein synthetic responses between pre- and postmenopausal females. We observed that postmenopausal females have an approximately 37% elevation in postabsorptive myofibrillar protein synthesis rates compared with their premenopausal counterparts (Fig. 8). This result suggests that postmenopausal females exhibit an elevated basal protein metabolism that may interfere with their muscles’ ability to respond robustly to the anabolic action of protein feeding and/or resistance exercise. Indeed, there is some indirect evidence for this assertion that postmenopausal females may benefit from hormone replacement therapy to restore the anabolic sensitivity to resistance exercise (49) and/or higher amounts of dietary protein (i.e., 35 g > 15 g) to elicit a robust postprandial myofibrillar protein synthetic response, albeit during energy restriction (42). However, it has been recently suggested that protein overconsumption (in excess of public health and nutrition recommendations) may have unintended consequences on human health (50), especially when exercise is not possible. Nevertheless, more work is required to underpin how to counteract this, albeit mild, hyper-protein metabolic basal state to restore muscle anabolic sensitivity.

We also measured key anabolic signaling proteins to provide additional insight into the regulation of myofibrillar protein synthesis after resistance exercise and protein ingestion in postmenopausal females. We previously showed that both total and phosphorylated YAP on Ser127 were increased after an acute bout of resistance exercise in younger adults (28). In contrast, in the current study, we demonstrated that resistance exercise did not modulate YAP at any time point during the recovery period in postmenopausal females. Similarly, mTORC1-dependent signaling events, as it relates to p70S6K, were not impacted by feeding and resistance exercise in postmenopausal females. Hence, it could be speculated that mechanical signals are not being optimally “sensed” in the muscle of postmenopausal females.

Our design also allows us to comment and contribute to the muscle protein field from a methodological perspective. First, the single biopsy approach has been routinely applied to provide an estimate of basal muscle protein turnover in young adults (32, 51) and older men (35). The basis of this approach is that background tracer enrichment of an easily assessable tissue (e.g., blood, skin, hair, etc.) mirrors that of skeletal muscle tissue (34), thereby minimizing the number of invasive muscle biopsies required to calculate basal muscle protein synthesis rates. It is assumed that the nonsteady state conditions within the intracellular free amino acid pool at the onset of the infusion are quite trivial provided the duration of the lead-in infusion before the initial biopsy is of sufficient duration (≥ 3 h) (35). Here, we provide confirmation that the single biopsy approach can reliably estimate basal muscle protein synthesis for older women, a population with elevated basal muscle protein synthesis rates when compared with other populations (young adults or older men; Fig. 8). In addition, we demonstrate that performing unilateral exercise does not modulate the postabsorptive myofibrillar protein synthesis in the contralateral nonexercised control leg (Fig. 7*A*; WATER condition). In other words, the acts of transitioning from a bed to an exercise machine(s) and returning to bed did not impact the anabolic response in the nonexercised leg. Finally, repeated muscle biopsies from the same muscle did not affect postabsorptive measurements of myofibrillar protein synthesis rates at 0–2 h, 2–4 h, or 0–4 h (Fig. 7*A*). This is consistent with other works that have shown a similar result with the mixed muscle protein synthetic response (52).

In conclusion, we report that resistance exercise alone or combined with feeding results in a differential temporal stimulation of myofibrillar protein synthesis rates that does not manifest into elevated cumulative myofibrillar protein synthetic response. This highlights that postmenopausal muscle is resistant to these potent anabolic stimuli, which may be underpinned by dysregulated basal myofibrillar protein synthesis rates when compared with their younger counterparts. These results suggest that higher volume load and increased nutritional intake may be necessary to counteract the anabolic resistance in postmenopausal females.

## DATA AVAILABILITY

Data will be made available upon reasonable request.

## SUPPLEMENTAL DATA

10.6084/m9.figshare.25215233 Supplemental Table S1: https://doi.org/10.6084/m9.figshare.25215233.

## DISCLOSURES

R.J. and M.P. are inventors of the international patent application WO 2019/20463 and have not been involved in the data collection or analysis. None of the other authors has any conflicts of interest, financial or otherwise, to disclose.

## AUTHOR CONTRIBUTIONS

R.J., M.P., and N.A.B. conceived and designed research; C.F.M., A.T.A., K.J.M.P., A.F.S., S.A.P., J.W.B., and N.A.B. performed experiments; C.F.M., A.T.A., H.-Y.F., A.V.U., Z.L., J.W.B., and N.A.B. analyzed data; C.F.M., A.T.A., and N.A.B. interpreted results of experiments; A.T.A. prepared figures; C.F.M., A.T.A., and N.A.B. drafted manuscript; C.F.M., A.T.A., K.J.M.P., H.-Y.F., S.A.P., J.W.B., R.J., M.P., and N.A.B. edited and revised manuscript; C.F.M., A.T.A., K.J.M.P., A.F.S., H.-Y.F., A.V.U., Z.L., S.A.P., J.W.B., R.J., M.P., and N.A.B approved final version of manuscript.
